# Supplementary material for: Confidence reports in decision-making with multiple alternatives violate the Bayesian confidence hypothesis
Source: Nat Commun. 2020 Apr 24;11:2004. doi: 10.1038/s41467-020-15581-6 (PMC7181620; doi:10.1038/s41467-020-15581-6)
Supplement: Supplementary file 1 — Supplementary Information [file 41467_2020_15581_MOESM1_ESM.pdf]

Supplementary Information

Supplementary Text

In addition to the three main models and their reduced versions discussed in the main text, we considered two types of alternative models. Alternative models of the first type are still Bayesian in the sense that the posterior probabilities of the categories are computed, but they contain different sources of variability than those considered in the main text. In alternative models of the second type (heuristic models), posterior probabilities are not computed; observers' category decisions and confidence reports are instead based on ad-hoc functions of the noisy measurement **x** and the stimuli. All the alternative models we tested contain a lapse rate as a free parameter. The model-fitting procedures are described in the main text (see **Methods**).

Bayesian models with different sources of variability

Sampling noise. Some studies have suggested that the brain approximates posterior probabilities by Monte Carlo sampling or simulation[1, 2, 3]. On each trial, the brain has access to n samples drawn from a multinomial distribution with parameters given by the true posterior probabilities, $\mathbf{p}=(p_1, p_2, p_3)=(p(C=1|\mathbf{x}), p(C=2|\mathbf{x}), p(C=3|\mathbf{x}))$. The probability mass function of this multinomial distribution is $f(n_1,n_2,n_3;n,\mathbf{p})=\frac{n!}{n_1!n_2!n_3!}p_1^{n_1}p_2^{n_2}p_3^{n_3}$, in which n_i represents the number of samples drawn from the ith category and $\sum_{i=1}^{3} n_i = n$. The number of samples n is a free parameter.

We assume that the observer's category decisions and confidence reports are based on the noisy posterior $\mathbf{q}=(q_1, q_2, q_3)$, where $q_i=\frac{n_i}{n}$. Thus, sampling noise is a form of decision noise, and the higher n, the less decision noise there is.

We took the Max, Difference and Entropy models (with both sensory noise and decision noise) and replaced the Dirichlet decision noise by sampling noise while keeping all the other aspects of the models unchanged. We named the resulting models Max-Sen-Samp, Diff-Sen-Samp and Ent-Sen-Samp. We also tested versions of the models that do not include sensory noise (Max-Samp, Diff-Samp and Ent-Samp models).

Noisy measurement of the category mean. In the three main models (Max, Difference and Entropy), given a uniform prior and the assumption that the observer has perfect

knowledge about the stimulus distribution, the posterior probability of category C given the measurement of the target location $\mathbf{x}$ is $p(C|\mathbf{x}) \propto p(\mathbf{x}|C) = N(\mathbf{x};\mathbf{m}_c,(\sigma_s^2+\sigma^2)\mathbf{I})$ (see **Methods**). Here, we allow for the possibility that the category mean $\mathbf{m}_c$ is not known exactly but measured in a noisy fashion. Then, the posterior probability of category C is computed as $p(C|\mathbf{x},\hat{\mathbf{m}}_c) \propto N(\mathbf{x};\hat{\mathbf{m}}_c,(\sigma_s^2+\sigma^2)\mathbf{I})$, in which $\hat{\mathbf{m}}_c \sim N(\mathbf{m}_c,\sigma_m^2\mathbf{I})$ is the measurement of the mean of category C. σ_m is a free parameter that controls the amount of noise in the measurement of the category mean. We assume that across three categories, this measurement noise is identical and independent.

We took the Max, Difference and Entropy models (with both sensory noise and decision noise) and replaced the Dirichlet decision noise by noise in measuring the category mean while keeping all the other aspects of the models unchanged. The resulting models are named Max-Sen-Mean, Diff-Sen-Mean and Ent-Sen-Mean. We also tested versions of the models that only include noise in measuring the category mean (Max-Mean, Diff-Mean, Ent-Mean models), without sensory noise or Dirichlet decision noise.

Heuristic models

Distance model. This model makes decisions based on the distance from the measurement to the center of each category. The observer chooses the category with the shortest distance $\hat{C} = \underset{i}{\operatorname{argmin}}\, d_i$, in which $d_i = \|\mathbf{x}_i - \mathbf{m}_i\|$ is the distance between the measurement and the center of category i. We further assume that confidence depends on the difference between the two shortest distances. For example, if $d_1 < d_2 < d_3$, an internal confidence variable is computed as $c^* = d_2 - d_1$. The internal confidence variable c^* is then converted to a four-point confidence report c by applying three criteria b_1, b_2 and b_3.

Weighted-distance model. This model is similar to the distance model, except that the continuous confidence variable is a linear function of the distance to each group. For example, if $d_1 < d_2 < d_3$, the continuous confidence variable is computed as $c^* = -0.5d_1 + ad_2 + bd_3$, in which a and b are free parameters representing the weights for the medium and the longest distance respectively. The weight for the shortest distance is fixed at -0.5. Allowing the weights of all three distances to be free parameters would have been redundant: infinitely many combinations of the weights and the three criteria (b_1, b_2 and b_3) would have produced the same model predictions. Choosing a=0.5 and b=0 reduces the Weighted-distance model to the Distance model.

Distance-to-Boundary model. This model is inspired by the finding by Kepecs et al. (2008) that behavioral and neural correlates of confidence showed responses that varied as a function of the distance between the target and the category boundary in the stimulus space. As in the Distance model, to make category decisions, the observer chooses the category that is closest to the measurement: $\hat{C} = \underset{i}{\text{argmin}}\, d_i$. The internal confidence variable c^* is computed as the distance between the measurement $\mathbf{x}$ and a decision boundary. This decision boundary is defined as a line perpendicular to and goes through the midpoint of the line connecting the centers of the two nearest categories.

We tested three versions of each of the heuristic models above. A version that only considers the sensory noise σ^2 (Dist-Sen, DistW-Sen and Bound-Sen models), a version that only considers the noisy estimate of the category center modeled by σ_m (Dist-Mean, DistW-Mean and Bound-Mean models), and a version that considers both (Dist-Sen-Mean, DistW-Sen-Mean and Bound-Sen-Mean models).

Supplementary Tables

	#	Experiment 1	Experiment 2	Experiment 3
Diff-Sen-Dir	6	0 [-]	0 [-]	0 [-]
Max-Sen-Dir	6	391 [222, 569]	541 [371, 735]	100 [46, 156]
Ent-Sen-Dir	6	1937 [1363, 2562]	1631 [1179, 2159]	1113 [817, 1447]
Diff-Dir	5	121 [48, 199]	132 [30, 237]	36 [3, 77]
Max-Dir	5	440 [276, 621]	616 [421, 816]	113 [48, 176]
Ent-Dir	5	1913 [1314, 2544]	1683 [1208, 2198]	1092 [797, 1395]
Diff-Sen	5	737 [590, 914]	921 [664, 1196]	1171 [982, 1363]
Max-Sen	5	1504 [1217, 1792]	1223 [933, 1520]	2011 [1719, 2299]
Ent-Sen	5	4114 [3394, 4920]	2190 [1658, 2733]	3835 [3356, 4287]
Diff-Samp	5	154 [73, 238]	292 [162, 426]	122 [54, 194]
Max-Samp	5	581 [426, 753]	812 [573, 1047]	325 [248, 403]
Ent-Samp	5	1744 [1219, 2282]	1540 [1068, 2053]	1134 [911, 1413]
Diff-Sen-Samp	6	-25 [-65, 17]	91 [2, 198]	-5 [-51, 43]
Max-Sen-Samp	6	411 [242, 574]	573 [397, 754]	207 [128, 308]
Ent-Sen-Samp	6	1751 [1225, 2325]	1503 [1034, 1983]	1121 [877, 1376]
Diff-Mean	5	183 [13, 340]	42 [-124, 182]	263 [115, 392]
Max-Mean	5	507 [242, 768]	535 [337, 703]	352 [165, 533]
Ent-Mean	5	2607 [1955, 3335]	1730 [1272, 2239]	2236 [1778, 2716]
Diff-Sen-Mean	6	29 [-102, 161]	-65 [-190, 76]	132 [1, 245]
Max-Sen-Mean	6	429 [183, 686]	420 [259, 561]	292 [106, 480]
Ent-Sen-Mean	6	2632 [1975, 3356]	1677 [1200, 2178]	2254 [1806, 2741]
Dist-Sen	5	1749 [1350, 2169]	1381 [1174, 1587]	2069 [1808, 2321]
Dist-Mean	5	745 [496, 1029]	265 [109, 421]	759 [492, 1030]
Dist-Sen-Mean	6	713 [434, 1026]	179 [68, 312]	730 [470, 975]
DistW-Sen	7	829 [667, 976]	1014 [757, 1266]	1204 [991, 1428]
DistW-Mean	7	361 [179, 566]	103 [-63, 281]	455 [256, 666]
DistW-Sen-Mean	8	269 [130, 403]	16 [-103, 132]	344 [208, 495]
Bound-Sen	5	1464 [1127, 1847]	2305 [1784, 2762]	1726 [1196, 2231]
Bound-Mean	5	1829 [1435, 2300]	1566 [1178, 1954]	1834 [1388, 2206]
Bound-Sen-Mean	6	1306 [995, 1701]	1492 [1085, 1863]	1354 [944, 1735]
Ratio-Sen-Dir	6	25 [-19, 62]	-19 [-54, 15]	51 [18, 90]

Supplementary Table 1. ΔAIC of each model and experiment, computed as the AIC of each model minus the AIC of the Diff-Sen-Dir model (the Difference model with both the sensory noise and Dirichlet decision noise). ΔAIC is computed for individual participants and then summed across participants. The first two columns are the model name, and the number of the free parameters. For each model and experiment, the group-summed ΔAIC and bootstrapped 95% confidence interval are reported. Names of the model are denoted as decision rules paired with the sources of variability separated by hyphens (-). Diff: Difference model; Max: Max model; Ent: Entropy model; Dist: Distance model; DistW: Weighted distance model; Bound: Distance-to-bound model; Ratio: Ratio model; Sen: sensory noise; Dir: Dirichlet decision noise; Samp: Sampling noise; Mean: noisy estimation of category mean.

	#	Experiment 1	Experiment 2	Experiment 3
Diff-Sen-Dir	6	0 [-]	0 [-]	0 [-]
Max-Sen-Dir	6	391 [224, 574]	541 [372, 728]	100 [45, 155]
Ent-Sen-Dir	6	1937 [1356, 2569]	1631 [1182, 2142]	1113 [796, 1433]
Diff-Dir	5	72 [-3, 150]	85 [-21, 195]	-10 [-44, 33]
Max-Dir	5	390 [225, 553]	570 [361, 774]	67 [3, 133]
Ent-Dir	5	1863 [1275, 2509]	1637 [1191, 2142]	1046 [725, 1371]
Diff-Sen	5	688 [534, 859]	875 [615, 1147]	1125 [941, 1317]
Max-Sen	5	1455 [1156, 1749]	1176 [887, 1480]	1965 [1684, 2246]
Ent-Sen	5	4065 [3349, 4899]	2143 [1627, 2710]	3789 [3340, 4238]
Diff-Samp	5	104 [24, 191]	245 [110, 373]	76 [6, 146]
Max-Samp	5	531 [376, 684]	765 [526, 1001]	280 [206, 354]
Ent-Samp	5	1695 [1209, 2248]	1493 [990, 2023]	1088 [851, 1347]
Diff-Sen-Samp	6	-25 [-66, 18]	91 [2, 194]	-5 [-52, 46]
Max-Sen-Samp	6	411 [243, 589]	573 [411, 761]	207 [126, 306]
Ent-Sen-Samp	6	1751 [1226, 2277]	1503 [1047, 2013]	1121 [894, 1384]
Diff-Mean	5	133 [-38, 293]	-4 [-161, 130]	217 [76, 353]
Max-Mean	5	457 [194, 712]	489 [300, 652]	306 [112, 502]
Ent-Mean	5	2557 [1904, 3278]	1683 [1204, 2169]	2190 [1724, 2681]
Diff-Sen-Mean	6	29 [-103, 163]	-65 [-199, 74]	132 [5, 251]
Max-Sen-Mean	6	429 [180, 680]	420 [245, 564]	292 [105, 482]
Ent-Sen-Mean	6	2632 [1961, 3368]	1677 [1196, 2187]	2254 [1777, 2756]
Dist-Sen	5	1700 [1276, 2105]	1334 [1109, 1538]	2023 [1753, 2266]
Dist-Mean	5	695 [425, 978]	219 [59, 384]	713 [426, 985]
Dist-Sen-Mean	6	713 [439, 1014]	179 [68, 320]	730 [473, 975]
DistW-Sen	7	879 [723, 1023]	1060 [821, 1319]	1250 [1013, 1475]
DistW-Mean	7	411 [225, 600]	149 [-23, 333]	501 [304, 699]
DistW-Sen-Mean	8	369 [233, 505]	109 [-5, 226]	436 [295, 587]
Bound-Sen	5	1415 [1085, 1775]	2259 [1793, 2738]	1680 [1146, 2180]
Bound-Mean	5	1779 [1404, 2238]	1520 [1142, 1900]	1789 [1365, 2175]
Bound-Sen-Mean	6	1306 [980, 1678]	1492 [1102, 1871]	1354 [949, 1734]
Ratio-Sen-Dir	6	25 [-15, 62]	-19 [-55, 18]	51 [16, 93]

Supplementary Table 2. ΔBIC of each model and experiment, computed as the BIC of each model minus the BIC of the Diff-Sen-Dir model (the Difference model with both the sensory noise and Dirichlet decision noise). ΔBIC is computed for individual participants and then summed across participants. The first two columns are the model name, and the number of the free parameters. For each model and experiment, group-summed ΔAIC and bootstrapped 95% confidence interval are reported. Names of the model are denoted as decision rules paired with the sources of variability separated by hyphens (-). Diff: Difference model; Max: Max model; Ent: Entropy model; Dist: Distance model; DistW: Weighted distance model; Bound: Distance-to-bound model; Ratio: Ratio model; Sen: sensory noise; Dir: Dirichlet decision noise; Samp: Sampling noise; Mean: noisy estimation of category mean.

	#	Experiment 1	Experiment 2	Experiment 3
ΔAIC				
Diff-Sen-Dir	6	0 [-]	0 [-]	0 [-]
Max-Sen-Dir	6	349 [203, 508]	547 [371, 736]	114 [58, 169]
Ent-Sen-Dir	6	1333 [935, 1776]	1436 [991, 1885]	875 [531, 1274]
Diff-Dir	5	116 [42, 197]	115 [41, 197]	20 [-2, 51]
Max-Dir	5	422 [270, 580]	589 [383, 794]	105 [51, 157]
Ent-Dir	5	1377 [965, 1898]	1424 [1002, 1876]	877 [532, 1244]
Diff-Sen	5	234 [152, 333]	252 [146, 375]	504 [393, 615]
Max-Sen	5	628 [450, 809]	632 [443, 859]	882 [716, 1046]
Ent-Sen	5	1818 [1369, 2330]	1555 [1062, 2103]	2182 [1929, 2423]
ΔBIC				
Diff-Sen-Dir	6	0 [-]	0 [-]	0 [-]
Max-Sen-Dir	6	349 [204, 519]	547 [362, 741]	114 [56, 174]
Ent-Sen-Dir	6	1333 [933, 1767]	1436 [977, 1898]	875 [522, 1237]
Diff-Dir	5	66 [-8, 151]	69 [-6, 149]	-26 [-48, 6]
Max-Dir	5	372 [218, 530]	543 [340, 743]	59 [5, 114]
Ent-Dir	5	1328 [916, 1828]	1378 [945, 1814]	831 [490, 1227]
Diff-Sen	5	184 [105, 279]	206 [100, 323]	458 [349, 580]
Max-Sen	5	578 [409, 760]	585 [410, 798]	836 [677, 1006]
Ent-Sen	5	1769 [1320, 2283]	1509 [1044, 2064]	2136 [1893, 2366]

Supplementary Table 3. ΔAIC and ΔBIC of the models when fitting the confidence reports alone.

Supplementary Figures

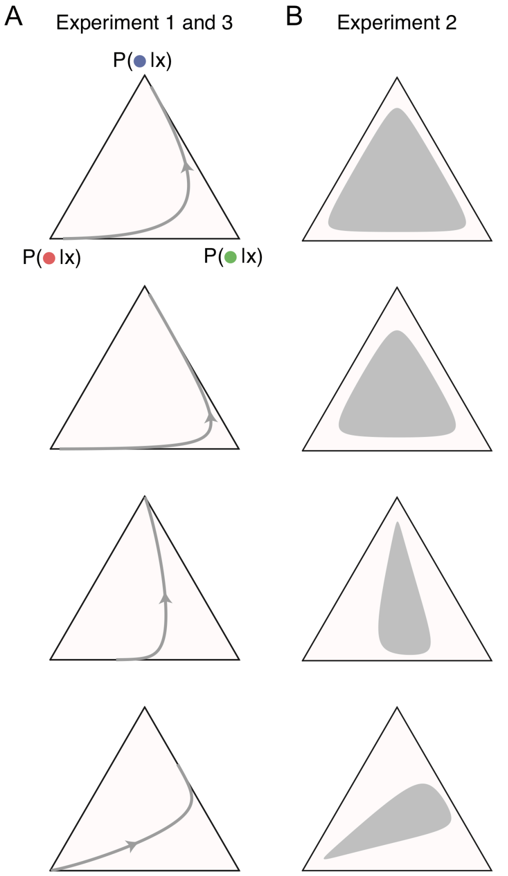

Supplementary Figure 1. Illustration of how observers' belief, posterior distribution, about the target category could change as a function of the target dot position. For illustration purpose, we consider a simplified case in which there is no sensory noise and no decision noise, so the posterior distribution only depends the target dot position and the distribution of each category. We use ternary plots to represent all possible posterior distributions. (A) Experiment 1 and 3: The four panels correspond to the four conditions depicted in Figure 1B. The gray lines and the arrows indicate the trajectory of the posterior distribution on the ternary plot as a target dot move from the left-end to the right-end of the screen. (B) Experiment 2: The four panels correspond to the four conditions depicted in Figure 1C. In the experiment, the target dot was uniformly sampled within a circle at the center of the screen with a radius of 2.6° (see Methods). All possible target dot locations within the circle correspond to a range of posterior probabilities indicated by the gray region in each panel.

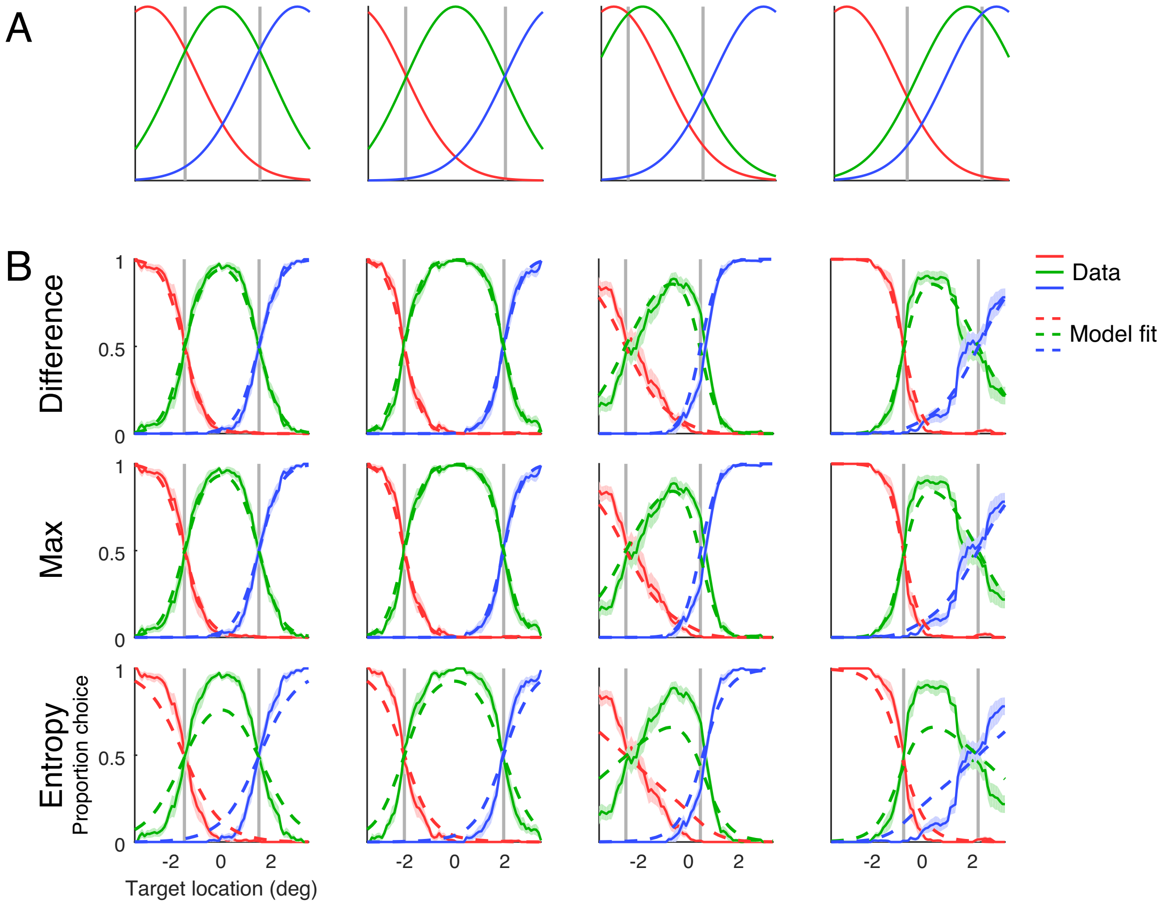

Supplementary Figure 2. Experiment 1. (A) Distribution of the reference dots in each condition. (B) The red (green, blue) lines represent the probability that the observers categorize the target dot to the red (green, blue) category as a function of the target dot location. Solid lines represent the group mean ± 1 s.e.m. The dashed lines represent the model fit averaged across individuals. In both (A) and (B), the gray vertical lines represent the boundary between two neighboring categories, the location where two neighboring categories have the same likelihood.

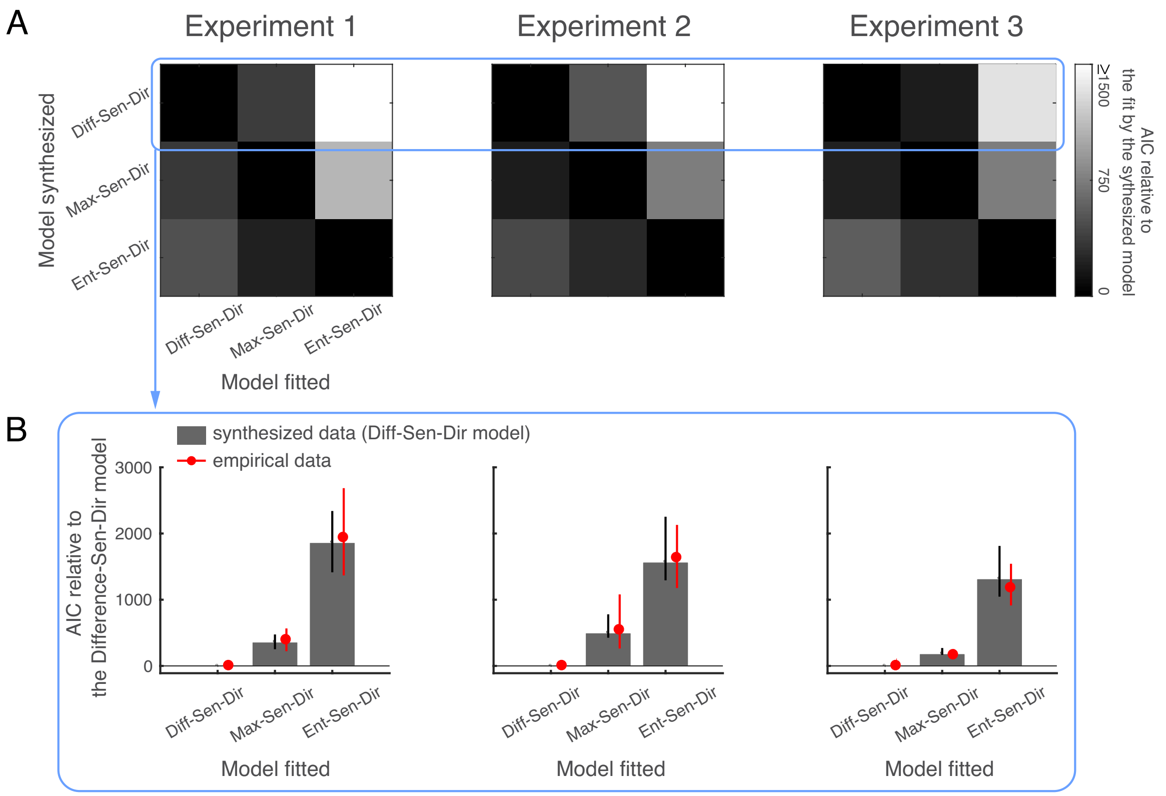

Supplementary Figure 3. Model recovery. (A) The darkness of the images represents ΔAIC (computed as the AIC of each fitted model minus the AIC of the fitted model that is the model used to synthesize the data) summed across participants. (B) The bars represent ΔAIC of the datasets synthesized based on the Difference model, corresponding to the top row of the images in (A). The red data points are the ΔAIC obtained in the experiments. Names of the model are denoted as decision rules paired with the sources of variability separated by hyphens (-). Diff: Difference model; Max: Max model; Ent: Entropy model; Sen: sensory noise; Dir: Dirichlet decision noise.

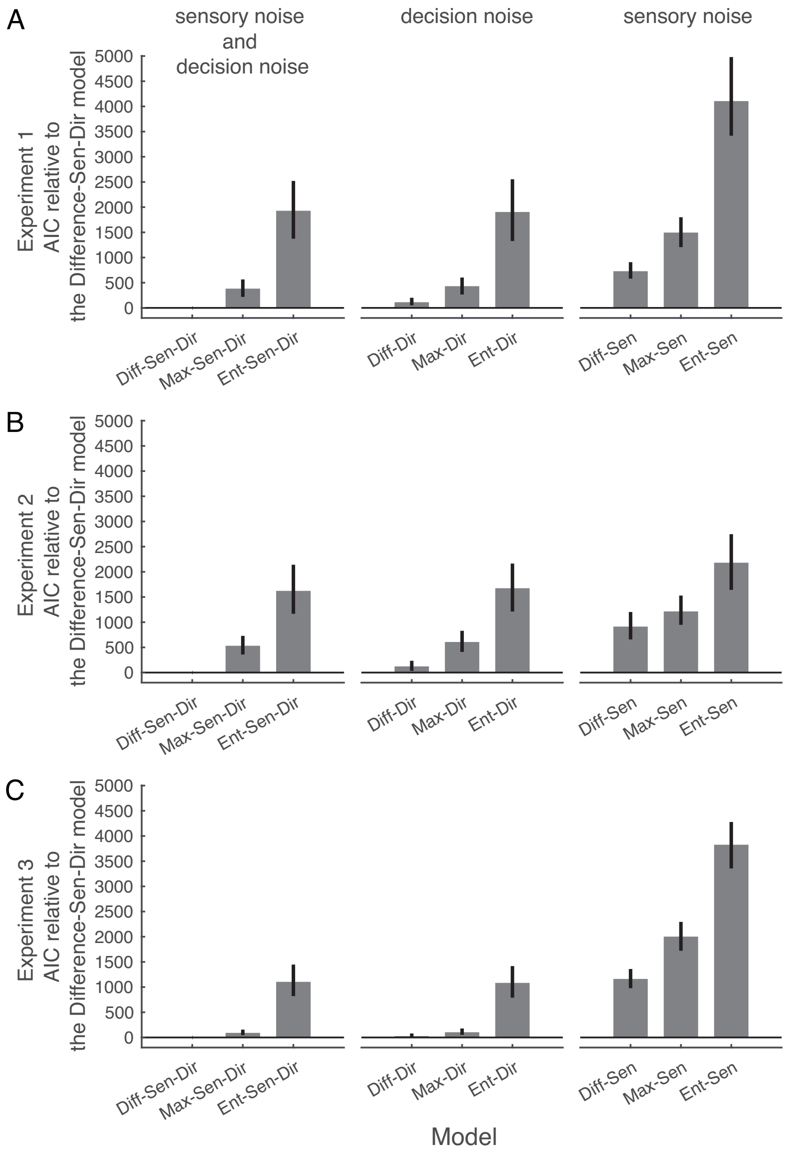

Supplementary Figure 4. Model comparison using AIC for both the full models (with both sensory and decision noise in the model) and the reduced models (with only the decision noise or only the sensory noise in the model). (A) Experiment 1. (B) Experiment 2. (C) Experiment 3. The bars represent ΔAIC (AIC of each model compared with the main Difference model, the Diff-Sen-Dir model) summed across participants. The error bars represent 95% bootstrapped confidence interval. Names of the model are denoted as decision rules paired with the sources of variability separated by hyphens (-). Diff: Difference model; Max: Max model; Ent: Entropy model; Sen: sensory noise; Dir: Dirichlet decision noise.

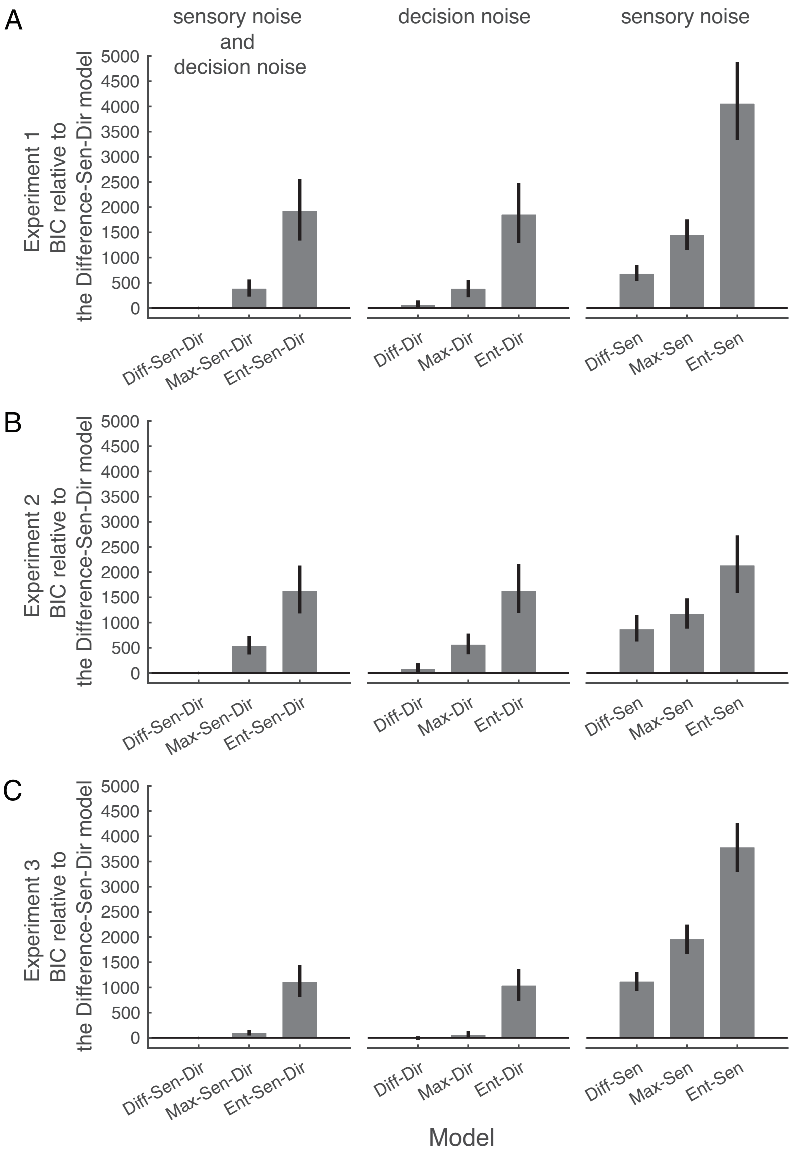

Supplementary Figure 5. Model comparison using BIC for both the full models (with both sensory and decision noise in the model) and the reduced models (with only the decision noise or only the sensory noise in the model). (A) Experiment 1. (B) Experiment 2. (C) Experiment 3. The bars represent ΔBIC (BIC of each model compared with the main Difference model, the Diff-Sen-Dir model) summed across participants. The error bars represent 95% bootstrapped confidence interval. Names of the model are denoted as decision rules paired with the sources of variability separated by hyphens (-). Diff: Difference model; Max: Max model; Ent: Entropy model; Sen: sensory noise; Dir: Dirichlet decision noise.

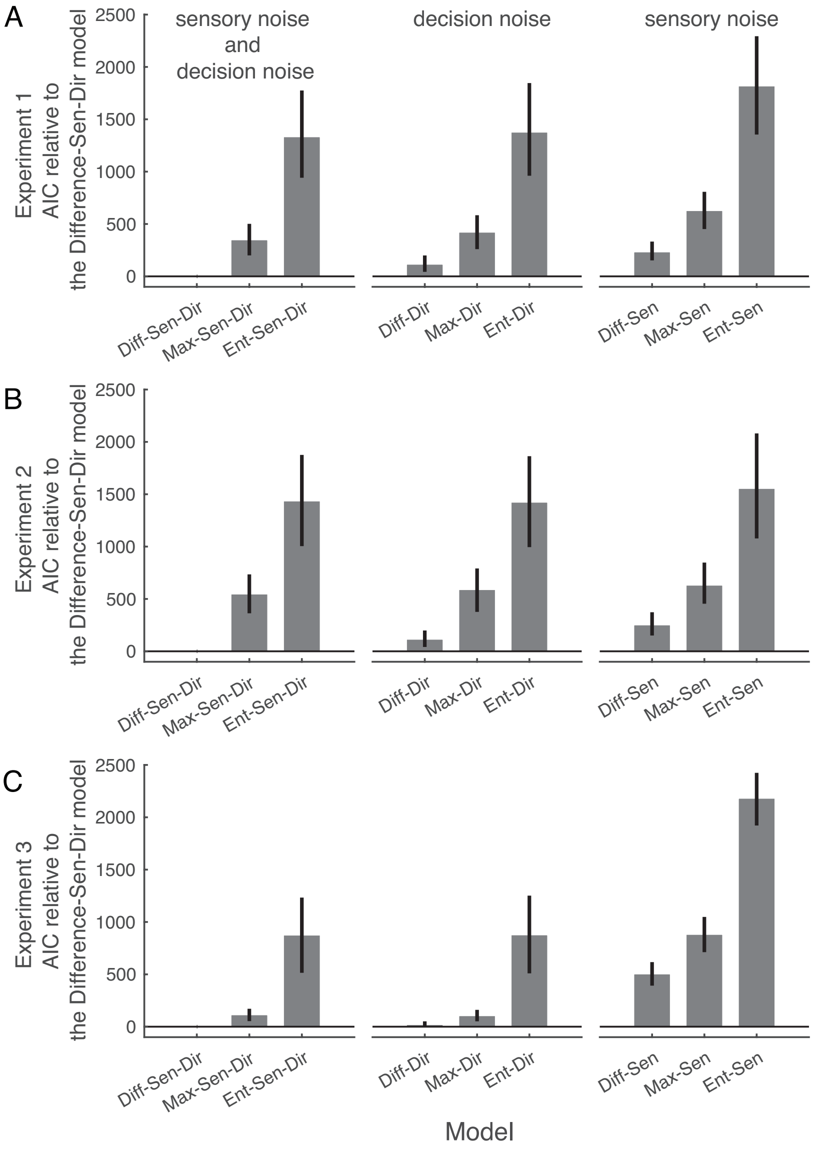

Supplementary Figure 6. Model comparison using confidence reports only. AIC for both the full models (with both sensory and decision noise in the model) and the reduced models (with only the decision noise or only the sensory noise in the model). (A) Experiment 1. (B) Experiment 2. (C) Experiment 3. The bars represent ΔAIC (AIC of each model compared with the main Difference model, the Diff-Sen-Dir model) summed across participants. The error bars represent 95% bootstrapped confidence interval. Names of the model are denoted as decision rules paired with the sources of variability separated by hyphens (-). Diff: Difference model; Max: Max model; Ent: Entropy model; Sen: sensory noise; Dir: Dirichlet decision noise.

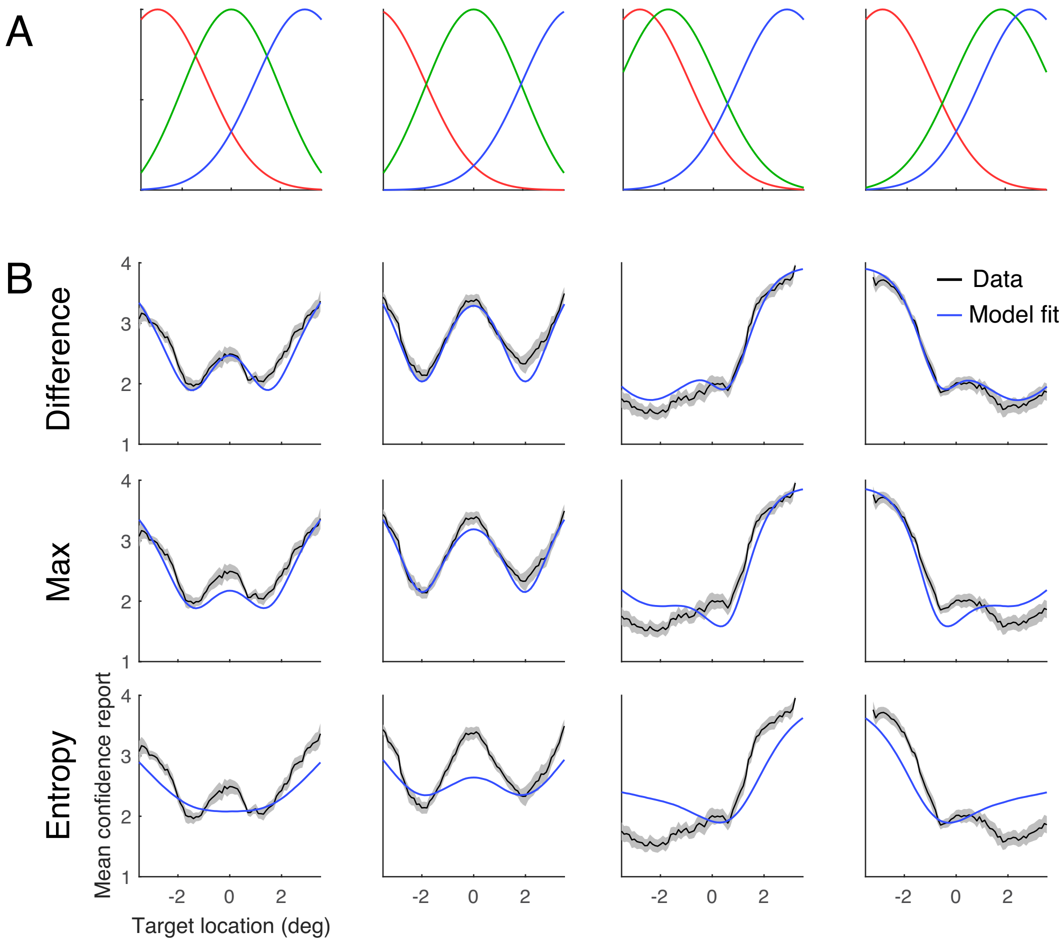

Supplementary Figure 7. Experiment 1. Model fit with confidence reports only, without jointly fitting the category decisions. (A) The distribution of the reference dots in each condition. (B) Mean confidence report as a function of target position for each of the four conditions. The black curves represent group mean ± 1 s.e.m. Blue curves represent the model fit averaged across individuals.

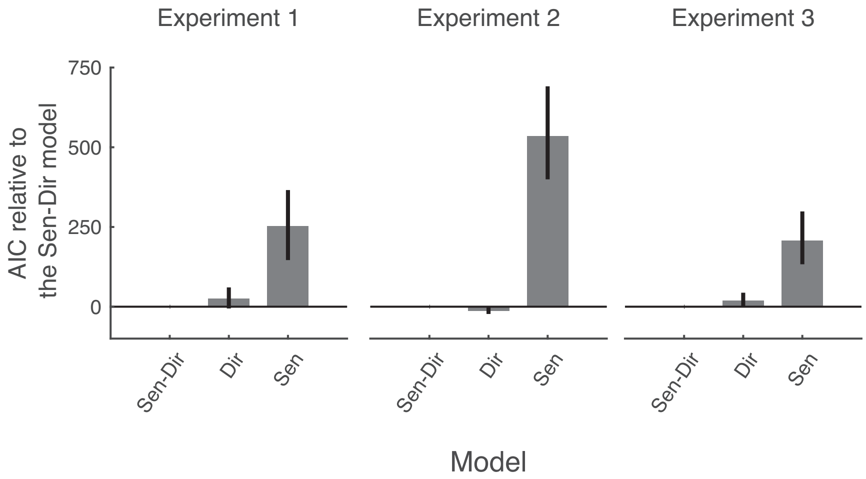

Supplementary Figure 8. Model comparison using category decisions only. Three models all choose the category with the highest posterior probability but consider different sources of variability: sensory and Dirichlet decision noise (Sen-Dir), Dirichlet decision noise only (Dir), and sensory noise only (Sen). The bars represent ΔAIC (AIC of each model compared with the Sen-Dir model) summed across participants. The error bars represent 95% bootstrapped confidence interval.

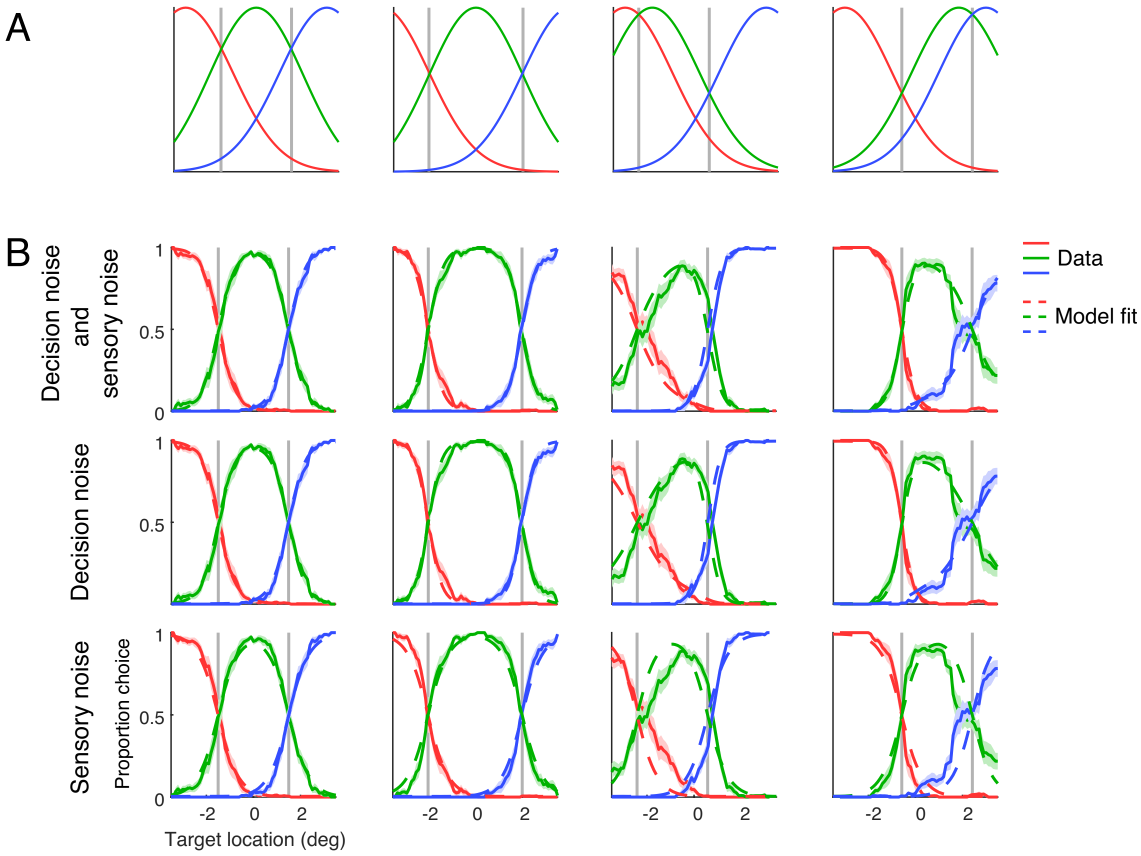

Supplementary Figure 9. Experiment 1. Model fit with category decisions only, without jointly fitting confidence reports. (A) Distribution of the reference dots in each condition. (B) The red (green, blue) lines represent the probability that the observers categorize the target dot to the red (green, blue) category as a function of the target dot location. Solid lines represent the group mean ± 1 s.e.m. The dashed lines represent the model fit averaged across individuals. In both (A) and (B), the gray vertical lines represent the boundary between two neighboring categories, the location where two neighboring categories have the same likelihood.

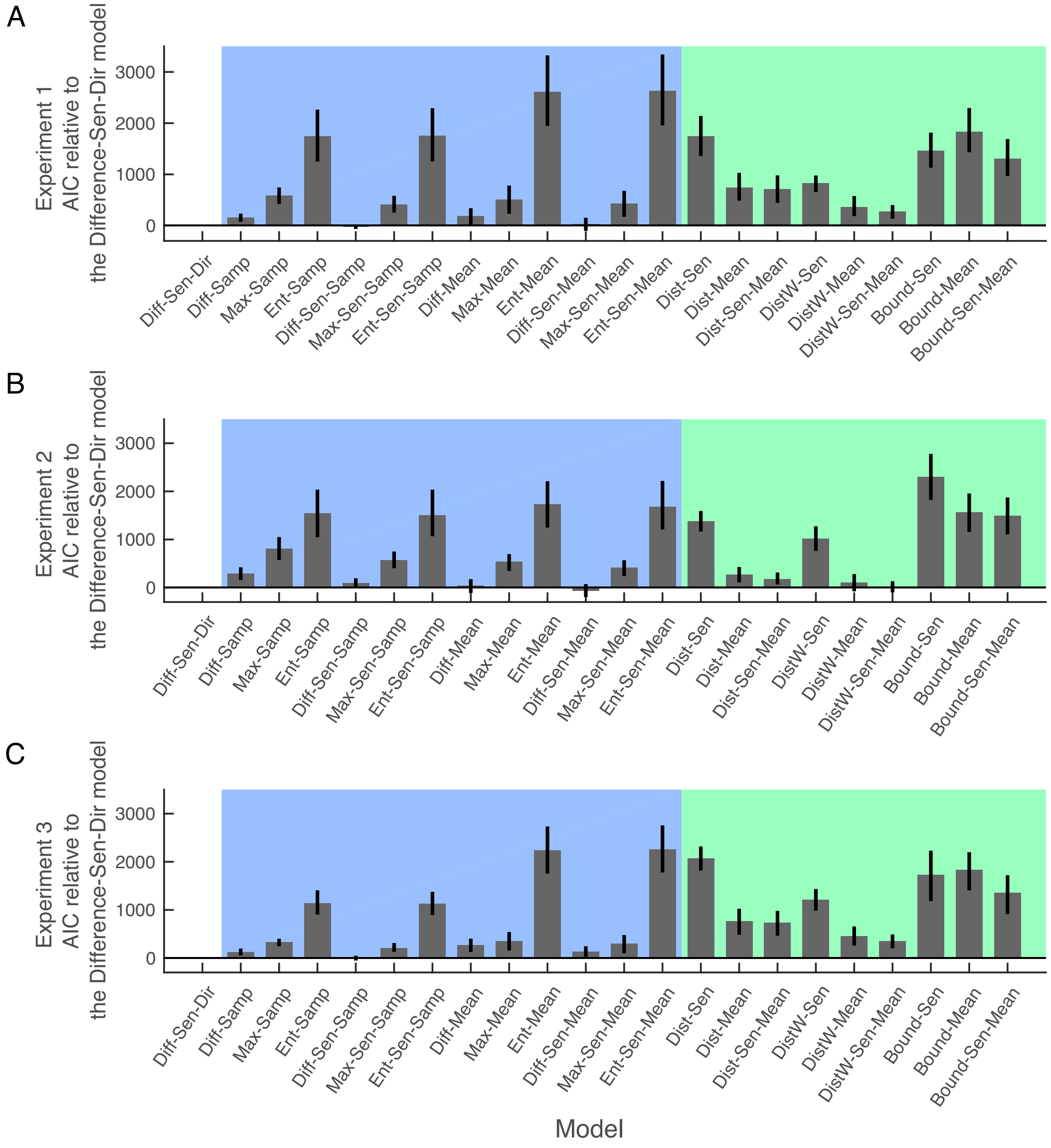

Supplementary Figure 10. Alternative models. Blue background: Bayesian models that compute posterior probabilities. Three confidence models (Difference, Max and Entropy) are paired with different sources of variability. Green background: Heuristic models that use the measurement of the target and the stimuli to perform the tasks. (A) Experiment 1. (B) Experiment 2. (C) Experiment 3. The bars represent ΔAIC (AIC of each model compared with the main Difference model, the Difference-Sen-Dir model) summed across participants. The error bars represent 95% bootstrapped confidence interval. Names of the model are denoted as decision rules paired with the sources of variability separated by hyphens (-). Diff: Difference model; Max: Max model; Ent: Entropy model; Dist: Distance model; DistW: Weighted distance model; Bound: Distance-to-bound model; Sen: sensory noise; Dir: Dirichlet decision noise; Samp: Sampling noise; Mean: noisy measurement of category mean.

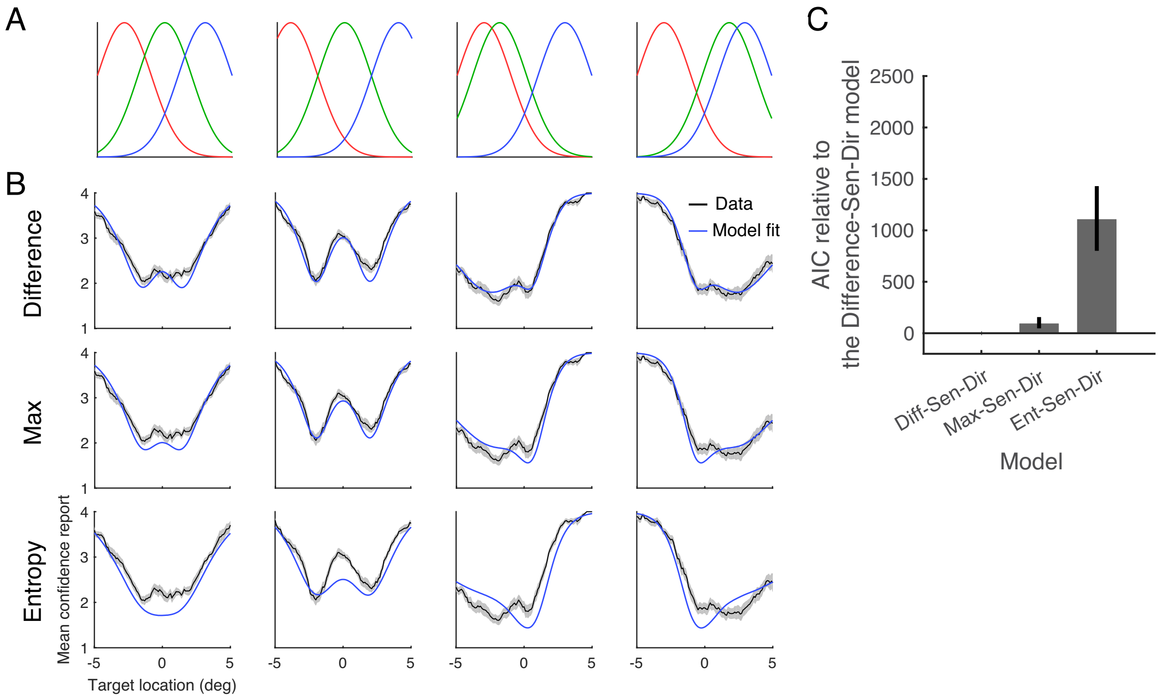

Supplementary Figure 11. Experiment 3. (A) The distribution of the reference dots in each condition. (B) Mean confidence report as a function of target position for each of the four conditions. The black curves represent group mean ± 1 s.e.m. Blue curves represent the model fit averaged across individuals. (C) Model comparisons using ΔAIC: AIC of each model compared with the Difference model. The bars represent ΔAIC summed across participants. The error bars represent 95% bootstrapped confidence interval.

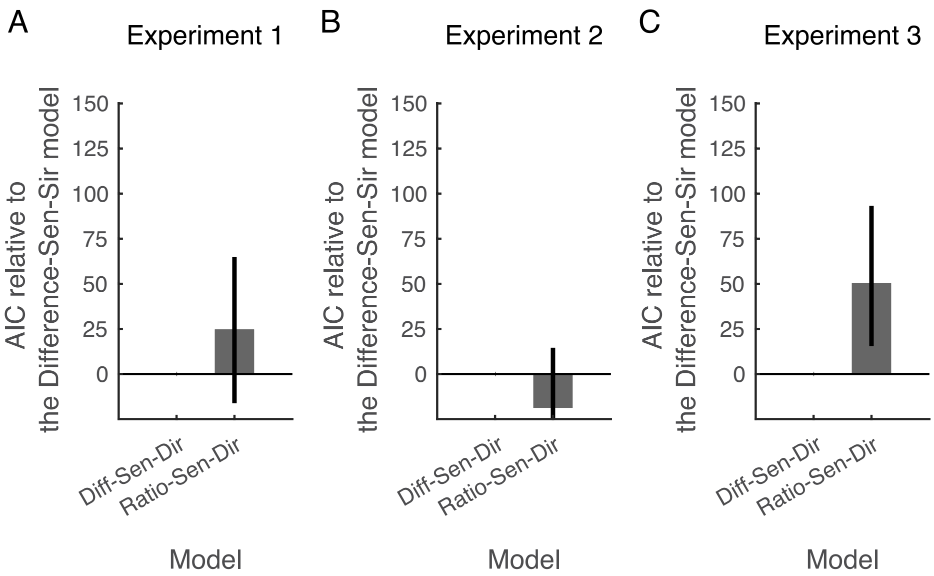

Supplementary Figure 12. Model comparison between the Difference model and the Ratio model using AIC. Sensory noise and Dirichlet decision noises are implemented in both models. (A) Experiment 1 (B) Experiment 2 and (C) Experiment 3. The bars represent ΔAIC (AIC of each model compared with the Difference model) summed across participants. The error bars represent 95% bootstrapped confidence interval..

Supplementary References

1. Shi L, Griffiths TL, Feldman NH, Sanborn AN. Exemplar models as a mechanism for performing Bayesian inference. *Psychonomic bulletin & review* **17**, 443-464 (2010).

2. Gershman SJ, Vul E, Tenenbaum JB. Multistability and perceptual inference. *Neural computation* **24**, 1-24 (2012).

3. Fiser J, Berkes P, Orbán G, Lengyel M. Statistically optimal perception and learning: from behavior to neural representations. *Trends in cognitive sciences* **14**, 119-130 (2010).
